# COVID-profiler: a webserver for the analysis of SARS-CoV-2 sequencing data

**DOI:** 10.1186/s12859-022-04632-y

**Published:** 2022-04-15

**Authors:** Jody Phelan, Wouter Deelder, Daniel Ward, Susana Campino, Martin L. Hibberd, Taane G. Clark

**Affiliations:** 1 Department of Infection Biology, Faculty of Infectious and Tropical Diseases, London School of Hygiene and Tropical Medicine, Keppel Street, London, WC1E 7HT UK; 2 Dalberg Advisors, 7 Rue de Chantepoulet, CH-1201 Geneva, Switzerland; 3 Faculty of Epidemiology and Population Health, London School of Hygiene and Tropical Medicine, London, WC1E 7HT UK

**Keywords:** NGS, Sequencing, Sars-Cov-2, Phylogenetics

## Abstract

**Background:**

SARS-CoV-2 virus sequencing has been applied to track the COVID-19 pandemic spread and assist the development of PCR-based diagnostics, serological assays, and vaccines. With sequencing becoming routine globally, bioinformatic tools are needed to assist in the robust processing of resulting genomic data.

**Results:**

We developed a web-based bioinformatic pipeline (“COVID-Profiler”) that inputs raw or assembled sequencing data, displays raw alignments for quality control, annotates mutations found and performs phylogenetic analysis. The pipeline software can be applied to other (re-) emerging pathogens.

**Conclusions:**

The webserver is available at http://genomics.lshtm.ac.uk/. The source code is available at https://github.com/jodyphelan/covid-profiler.

**Supplementary Information:**

The online version contains supplementary material available at 10.1186/s12859-022-04632-y.

## Background

During the last 20 years, there have been major infection outbreaks that have caused significant morbidity, including ‘spill-over events’ where a coronavirus has entered the human population from an animal host. The SARS-CoV-2 virus has caused the COVID-19 pandemic, with > 185 million individuals infected and > 4 million deaths across > 215 countries [1]. The small size of the viral genome (~ 30 kbp) allows for rapid and high throughput sequencing within health systems. The resulting data is being shared in the public domain (e.g. NCBI and GISAID), with large and growing numbers of genome sequences available (> 2 million) [2]. Phylogenetic analysis of sequence variation can provide insights into the spread and evolution of the virus, and a temporal analysis can reveal mutations that might affect diagnostic efficacy and lead to vaccine escape. Potential transmission networks to inform infection control can be revealed through matching genomes, and clade-defining mutations can be found. The detection of amino-acid changes leading to increased virus fitness, maintained by sweeps increasing mutation frequencies (“positive selection”), can reveal sites that are functionally important. Important genotype–phenotype associations (e.g., for virulence) may be identified through finding signatures of convergent evolution, where the causal mutation occurs independently in unrelated branches of the phylogenetic tree. Despite there being available tools for the online visualisation of SARS-CoV-2 virus genetic diversity [2, 3] and selection analysis [4], timely phylogenetic analysis is computationally difficult with large numbers of sequenced isolates. Further, infection control sequencing hubs urgently need bioinformatic pipelines to accurately process sequence data into mutations, providing insights into local circulating viruses and to inform on importation and diagnostic robustness. To enable the processing of sequencing data, we have designed command-line and webserver tools (“COVID-Profiler”) to automate the analysis, visualisation and profiling of SARS-CoV-2, thereby assisting COVID-19 control decision making.

## Implementation

The COVID-Profiler command-line tool has a number of functions, including variant characterisation, isolate profiling, primer design and evaluation, and phylogenetic analysis. For profiling, fastq files (compressed with gzip or uncompressed) are trimmed using trimmomatic [5] and aligned against the reference genome (NC_045512) using bwa-mem software [6]. Variant calling is performed using bcftools software [7]. User-defined depth thresholds can filter out variants. Remaining variants are annotated with their functional effect (e.g., gene, amino acid change) using a combination of bcftools csq and custom scripts, which account for the ribosomal slippage. A final genome is generated with bcftools consensus, and positions with low coverage [8] are masked. For fasta input, sequences are aligned to the reference using minimap2 [9] and variants are called with paftools.js software. Variant outputs are in files of vcf format. Users can aggregate data across several runs to create a table of mutations in text format. Primer conservation scores (e.g., for diagnostics) are calculated using blast, and an in-house script estimates the number of mismatches. Choropleth maps are rendered using plotly software. Sequence logo plots are generated using seqlogo software (github.com/betteridiot/seqlogo). Sequences are aligned using mafft software [10] and alignments are masked on non-coding ends. Phylogenetic trees are constructed using iq-tree software [11] with the best model found by iq-tree ModelFinder. The webserver allows for the online processing and profiling of uploaded isolate sequences. It is developed using the flask micro web framework. Users upload data in fastq or fasta format. All output files are available to download, and include identified mutations presented in a table format. Alignments (“bam” files) are visualised using pileup.js [12]. Mutations in those proteins that have been structurally characterised are visualised using a bio-pv.js (biasmv.github.io/pv/) library. Flowcharts depicting the bioinformatic pipeline and its inputs and outputs can be found in Additional file 1: Figure S1.

## Results

To highlight the utility of the tool, we sought to characterize the diversity in a large dataset of 561,002 sequences from the GISAID database. Sequences were selected using a random sample of isolates between 24th December 2019 and 6th July 2021 allowing a maximum of 20,000 isolates per country. Using COVID-Profiler, the sequences were aligned, and 59,940 SNPs were identified (42,734 non-synonymous; 17,206 synonymous changes). We then aimed to characterize whether this diversity affected the performance of PCR diagnostics used by public health authorities, and whether it has been influenced by selective pressure.

### Diagnostic performance

**Fig. 1:**
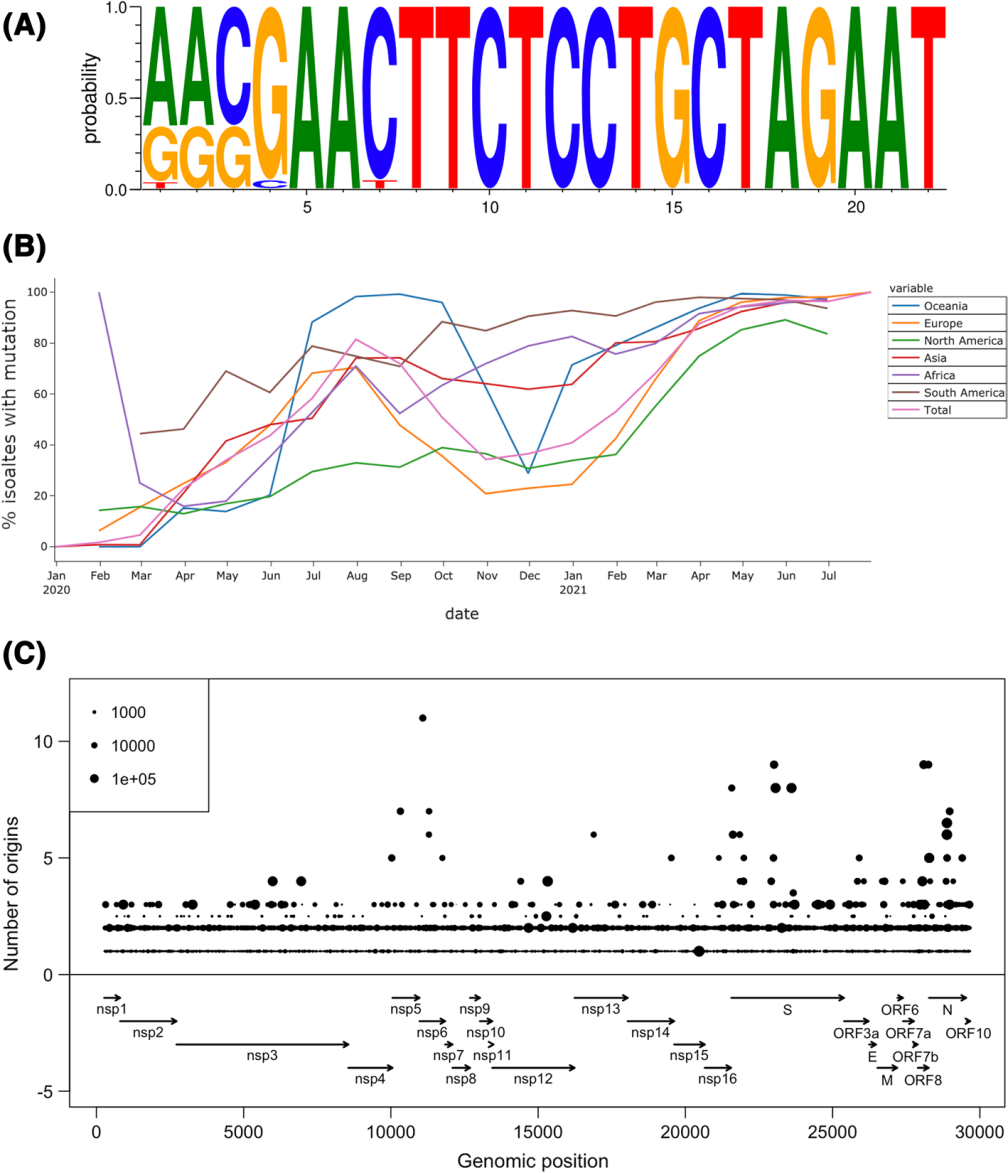
Results from the primer conservation tool. **A** A sequence logo plot indicating the location of mutations in the primer binding site (PBS). **B** Samples with mutations in PBSs across time and continent. **C** Convergent evolution analysis, where each genomic position is represented by a dot with the average number of origins indicated on the y-axis. The number of isolates containing the mutation is proportional to the size of the dot

Rapid and accurate detection of infections through diagnostics is central to infection control. We sought to characterise the conservation of primer binding sites (PBSs) of 12 PCR diagnostics applied worldwide (Additional file 1: Table S1). Analysis by COVID-Profiler revealed that eleven diagnostics had high conservation of PBSs, but one had a high proportion (75.1%) of isolates with >  = 1 mismatch present in the forward PBS. The sequence logo plot reveals the mismatches were located at the 5’ end, and thus might not have a severe effect on binding (Fig. 1a). The number of isolates with mutations in the PBSs was observed to increase in all continents during the timeline of the pandemic (Fig. 1b). The rapid increase in allele frequency could result from selective pressure, sampling bias or genetic drift effects.

### Selective pressure

COVID-Profiler was used to determine whether the mutations were under selective pressure. Specifically, the dataset was used to find convergent evolution, which is defined by multiple independent origin of a mutation in phylogenetic space and is a signature of selective pressure. Across 1,000 independent subsets of the data (N = 500), we counted the number of independent origins of mutations within each subset. Most mutations (n = 49,443, 82.5%), had an average occurrence of at most one (Fig. 1c). However, several mutations had substantially higher counts with the top 10 mutations having occurrences greater than 6. These include mutations in the genes *S* (E484K/Q, L5F, N501T and P681H/R), *N* (M234I), *nsp6* (L37F, F108L), *orf8* (L68*, F120L) and the 3C-like proteinase (L90R). Of the mutations found in the spike protein (*S*), E484K and N501T have been classified as variants of concern with a high confidence of conferring an antigenic change (http://sars2.cvr.gla.ac.uk/cog-uk/). Interestingly, the Nucleocapsid protein (*N*) M234I mutation is located in a position which has been found to have immunological significance both in epitope prediction studies as well as in-vitro epitope mapping experiments[13, 14] as well being linked to isolates with false negative antigenic tests [15]. These observations demonstrate the utility in the detection of convergent evolution and more broadly the analysis of genomic diversity and tools which support this analysis.

## Conclusions

The online COVID-Profiler tool will assist with the important analysis and profiling of SARS-CoV-2 viral sequences within clinical and infection control settings. With the vaccine rollout, it is important to use tools like COVID-Profiler to monitor mutations and the selective pressure driving their patterns of evolution. The tool can be extended to include additional functionality (e.g., assign strain types), as well as be modified for the analysis of other (re-) emerging pathogens.

## Supplementary Information

**Additional file 1**. **Table S1:** Primers and the number of samples with a mismatch in the different components. **Figure S1:** A UML figure representing the data flow and program calls used by covid-profiler.

## Data Availability

The datasets generated and/or analysed during the current study are available from https://www.gisaid.org/ and https://www.ncbi.nlm.nih.gov/sars-cov-2/

## Abbreviation

PBS: Primer binding site

## References

[1] World Health Organization. WHO COVID-19 dashboard. 2020. https://covid19.who.int/.

[2] Ward D, Higgins M, Phelan J, Hibberd M, Campino S, Clark T. An integrated in silico immuno-genetic analytical platform provides insights into COVID-19 serological and vaccine targets. bioRxiv. 2020;:2020.05.11.089409. doi:10.1101/2020.05.11.089409. 10.1186/s13073-020-00822-6 PMC7790334 33413610

[3] Hadfield J, Megill C, Bell SM, Huddleston J, Potter B, Callender C (2018). Nextstrain: real-time tracking of pathogen evolution. Bioinformatics.

[4] Datamonkey. Sars-Cov-2 Natural selection analysis. 2020. http://covid19.datamonkey.org/.

[5] Bolger AM, Lohse M, Usadel B (2014). Trimmomatic: a flexible trimmer for Illumina sequence data. Bioinformatics.

[6] Li H. Aligning sequence reads, clone sequences and assembly contigs with BWA-MEM. 2013. http://arxiv.org/abs/1303.3997. Accessed 6 Sep 2017.

[7] Li H (2011). A statistical framework for SNP calling, mutation discovery, association mapping and population genetical parameter estimation from sequencing data. Bioinformatics.

[8] Quinlan AR, Hall IM (2010). BEDTools: a flexible suite of utilities for comparing genomic features. Bioinformatics.

[9] Li H (2018). Minimap2: pairwise alignment for nucleotide sequences. Bioinformatics.

[10] Nakamura T, Yamada KD, Tomii K, Katoh K (2018). Parallelization of MAFFT for large-scale multiple sequence alignments. Bioinformatics.

[11] Minh BQ, Schmidt HA, Chernomor O, Schrempf D, Woodhams MD, von Haeseler A (2020). IQ-TREE 2: new models and efficient methods for phylogenetic inference in the genomic era. Mol Biol Evol.

[12] Vanderkam D, Aksoy BA, Hodes I, Perrone J (2016). Hammerbacher J. pileup.js: a JavaScript library for interactive and in-browser visualization of genomic data. Bioinformatics.

[13] Liang T, Cheng M, Teng F, Wang H, Deng Y, Zhang J (2021). Proteome-wide epitope mapping identifies a resource of antibodies for SARS-CoV-2 detection and neutralization. Signal Transduct Target Ther.

[14] Ward D, Higgins M, Phelan JE, Hibberd ML, Campino S, Clark TG (2021). An integrated in silico immuno-genetic analytical platform provides insights into COVID-19 serological and vaccine targets. Genome Med.

[15] Vecchio C Del, Brancaccio G, Brazzale AR, Lavezzo E, Onelia F, Franchin E, et al. Emergence of N antigen SARS-CoV-2 genetic variants escaping detection of antigenic tests. 10.1101/2021.03.25.21253802

