# Supplementary material for: COVID-profiler: a webserver for the analysis of SARS-CoV-2 sequencing data
Source: BMC Bioinformatics. 2022 Apr 15;23:137. doi: 10.1186/s12859-022-04632-y (PMC9012066; doi:10.1186/s12859-022-04632-y)

| Name | Forward Primer Global Mismatch % | Reverse Primer Global Mismatch % | Probe Global Mismatch  % |
| --- | --- | --- | --- |
| China_N^1^ | 74.11 | 50.60 | 0.30 |
| China_orf1ab^1^ | 0.34 | 0.45 | 0.28 |
| Germany_E^2^ | 0.25 | 0.07 | 0.20 |
| Germany_RdRP^2^ | 3.85 | 0.03 | 0.87 |
| Pasteur_E_Sarbeco^2^ | 0.25 | 0.07 | 0.12 |
| Pasteur_IP2^3^ | 0.07 | 1.93 | 0.20 |
| Pasteur_IP4^3^ | 0.24 | 0.64 | 4.53 |
| Hong_Kong_N^4^ | 0.99 | 1.42 | 1.28 |
| Hong_Kong_nsp14^4^ | 0.40 | 0.30 | 0.12 |
| USA_N1^5^ | 1.26 | 0.57 | 2.31 |
| USA_N2^5^ | 1.47 | 0.56 | 1.97 |
| USA_N3^5^ | 1.82 | 1.65 | 1.59 |

**Table S1**

**Primers and the number of samples with a mismatch in the different components**

1) <http://ivdc.chinacdc.cn/kyjz/202001/t20200121_211337.html>

2) <https://www.who.int/docs/default-source/coronaviruse/protocol-v2-1.pdf?sfvrsn=a9ef618c_2>

3) <https://www.who.int/docs/default-source/coronaviruse/real-time-rt-pcr-assays-for-the-detection-of-sars-cov-2-institut-pasteur-paris.pdf?sfvrsn=3662fcb6_2>

4) <https://www.who.int/docs/default-source/coronaviruse/peiris-protocol-16-1-20.pdf?sfvrsn=af1aac73_4>

5) <https://www.who.int/docs/default-source/coronaviruse/real-time-rt-pcr-assays-for-the-detection-of-sars-cov-2-institut-pasteur-paris.pdf?sfvrsn=3662fcb6_2>

**Figure S1**

**A UML figure representing the data flow and program calls used by COVID-Profiler**


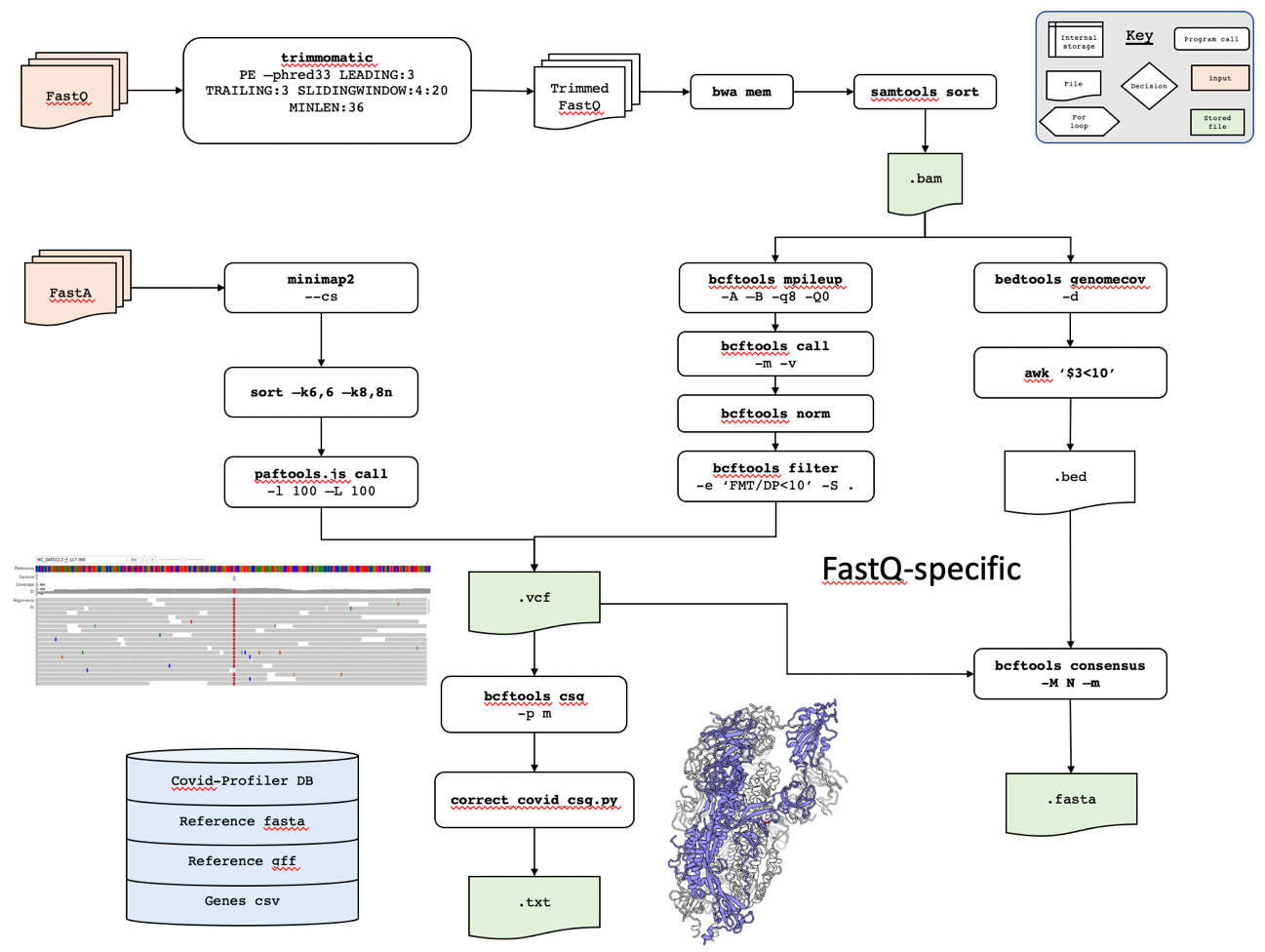


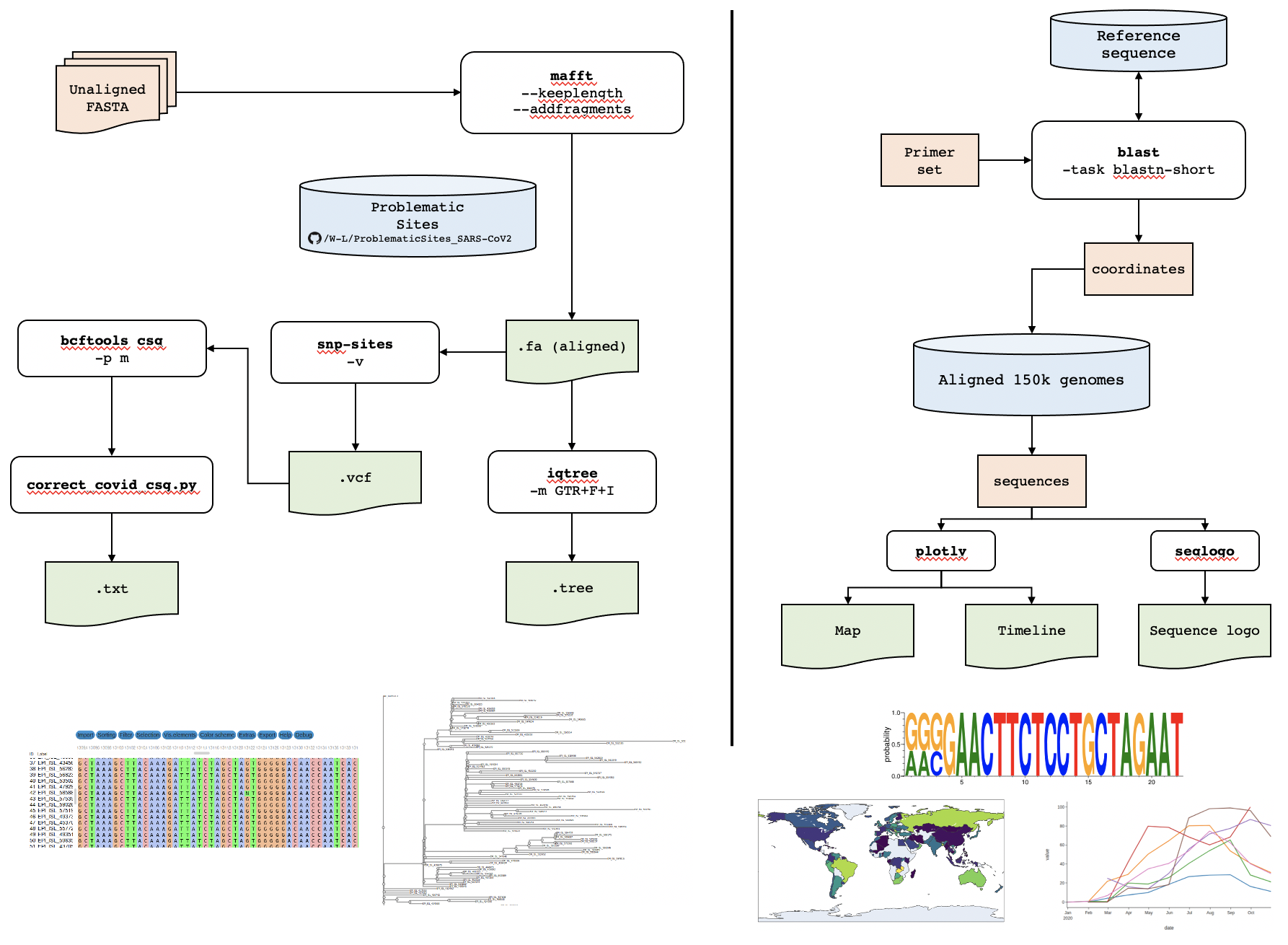

Supplement: Supplementary file 1 — Additional file 1. Table S1: Primers and the number of samples with a mismatch in the different components. Figure S1: A UML figure representing the data flow and program calls used by covid-profiler. [file 12859_2022_4632_MOESM1_ESM.docx]
